# Challenges in advanced heart failure care in Japan: Bridging the gap in durable mechanical circulatory support utilization and heart transplantation

**DOI:** 10.1016/j.jhlto.2024.100204

**Published:** 2024-12-31

**Authors:** Satoshi Miyashita, Francisco B. Alexandrino, Amanda R. Vest, Tomohiro Fujisaki, Wai Hong Wilson Tang, Yasumasa Tsukamoto, Koichiro Kinugawa

**Affiliations:** a Department of Cardiovascular Medicine Heart, Vascular, and Thoracic Institute Cleveland Clinic Foundation, Cleveland, Ohio; b Department of Internal Medicine, Primary Care Institute, Cleveland Clinic Foundation, Cleveland, Ohio; c Department of Cardiovascular Medicine, National Cerebral and Cardiovascular Center, Suita, Osaka, Japan; d Department of Transplantation, National Cerebral and Cardiovascular Center, Suita, Osaka, Japan; e The Second Department of Internal Medicine, University of Toyama, Toyama, Toyama, Japan

**Keywords:** heart transplant, left ventricular assist device, advanced heart failure, cardiogenic shock, heart failure

## Abstract

In Japan, the incidence of heart failure has escalated to become a major cause of morbidity and mortality. Advanced therapeutic interventions, such as heart transplantation and durable left ventricular assist devices, have become a focus of interest in recent decades. However, the unique sociocultural landscape of Japan, coupled with a historically controversial background of organ transplantation, has impeded progress in these areas. This has resulted in a notable donor shortage and the absence of a robust framework for heart transplantation. This review aims to highlight the specific challenges faced in advancing heart failure care in Japan and proposes strategic solutions to overcome these barriers.

## Background

More than 64 million people worldwide suffer from heart failure, including 6.7 million in the United States.^1^, ^2^ Japan, famous for its longevity—81.49 years for men and 87.60 years for women—has long been considered a paradigm of health.^3^ However, the growing prevalence of cardiovascular disease, particularly heart failure, is challenging this idealized image. Similar to many nations, Japan is also facing a heart failure epidemic, reflecting a global health challenge.^4^ An aging population coupled with high smoking rates (27.1% for men and 7.6% for women) and the adoption of Western dietary patterns are exacerbating the incidence of cardiovascular disease, particularly heart failure, in Japan.^5^, ^6^ A retrospective study showed that between 2014 and 2019, the prevalence of heart failure in Japan was 2.2% to 3.7% in individuals aged ≤74 years, reaching 6.5% in all age groups, with a steady increase in prevalence over those years.^7^ This condition places a significant burden on the Japanese health care system, characterized by prolonged hospital stays of 14 to 21 days, in-hospital mortality rates of 4.7% to 6.4%, 1-year readmission rates of 24% to 27%, and 1-year all-cause mortality rates of 18% to 22%.^8^

Advanced heart failure is characterized by severe, persistent symptoms and signs of heart failure despite maximal medical therapy, indicating the inability of the heart to maintain adequate circulation.^9^ In a large retrospective study from Japan that included 160,559 patients admitted for cardiogenic shock and requiring mechanical circulatory support, in-hospital mortality was 30.1% and only 0.64% of patients underwent advanced therapies: heart transplantation 0.04% and durable left ventricular assist device (LVAD) 0.6%.^10^

Heart transplantation and durable LVADs are the main treatment options for patients with advanced heart failure when clinically indicated. In the United States, 4,545 patients underwent heart transplantation in 2023, while 2,517 patients received durable LVAD implants in 2022.^11^, ^12^ In contrast, in Japan, despite the increasing number of heart transplants and durable LVAD implants, only 851 heart transplants and 1,381 permanent LVAD implants have been performed to date due to the country's unique and complex social and cultural background.^13^, ^14^ The purpose of this review is to elucidate the existing and future challenges in the field and to explore strategies for advancing the future treatment of advanced heart failure.

## History of advanced heart failure therapies in Japan

**Figure 1:**
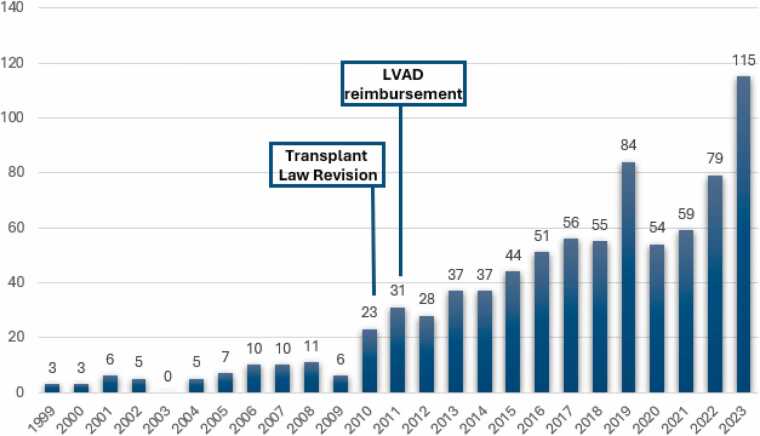
The annual number of heart transplants in Japan. The rate of heart transplants in Japan has increased but continues to be relatively low. LVAD, left ventricular assist device.

The first heart transplant, performed by Dr Juro Wada in 1968, was controversial for several reasons, including questionable indications for the surgery and the lack of clear confirmation of the donor’s brain death.^15^ As a result of the recipient’s death, Dr Wada was charged with murder. This tumultuous event catalyzed widespread ethical and procedural debates, culminating in an extended hiatus in heart transplant practice that lasted until 1997. The enactment of the Organ Transplant Law in 1997 allowed for brain-dead organ donation with written consent from the donor and family, but restricted donations from those under the age of 15, forcing many to seek transplants abroad. The landscape changed in 2010, following the Istanbul Declaration, when the revised organ transplant law allowed donations only with family consent, broadening the potential donor pool. However, as Figure 1 illustrates, while the number of heart transplants has gradually increased, reaching 115 cases in 2023, it remains relatively low compared to global standards. This reflects ongoing challenges, including donor shortages, cultural barriers, and systemic inefficiencies. In Japan, durable LVAD implantation was initially approved in 2011 as a bridge to transplantation. After a delay of nearly 10 years, a destination therapy LVAD was finally approved and reimbursed in Japan in May 2021. However, of the 1,381 patients with durable LVADs, only 3%, or 47 patients, received these devices as destination therapy.^14^, ^16^

## Critical imbalance in heart donation demand and supply in Japan

Heart failure in Japan is notably prevalent among elderly patients, who constitute a significant proportion of the heart failure population. Among these individuals, heart failure with preserved ejection fraction is more common than heart failure with reduced ejection fraction.^17^ Heart failure in elderly patients in Japan is often accompanied by a complex array of comorbidities, which complicate heart failure management and limit the suitability of these patients for advanced therapies that often preclude the elderly.^18^ In this context, optimizing heart failure care for this population is essential. Management should emphasize the initiation and adjustment of guideline–directed medical therapy. Additionally, comprehensive care must include social support systems, tailored rehabilitation programs, and patient and caregiver education, which can significantly improve functional status and quality of life. Even considering that heart failure predominantly affects the elderly in Japan, it remains prevalent across a wide range of age groups. Consequently, a substantial number of patients in Japan still require advanced therapies.

Japan faces a significant shortage in its organ donation pool, clearly evident from its low global donor rates. According to a 2022 report, Japan's donor rate stood at a mere 0.9 donors per million population (pmp), significantly lower compared to the United States, which reported a rate of 44.5 donors pmp. Other countries such as the United Kingdom and Germany also demonstrated higher rates with 21.08 and 10.34 donors pmp, respectively, while South Korea recorded a rate of 7.88 donors pmp.^19^

**Figure 2:**
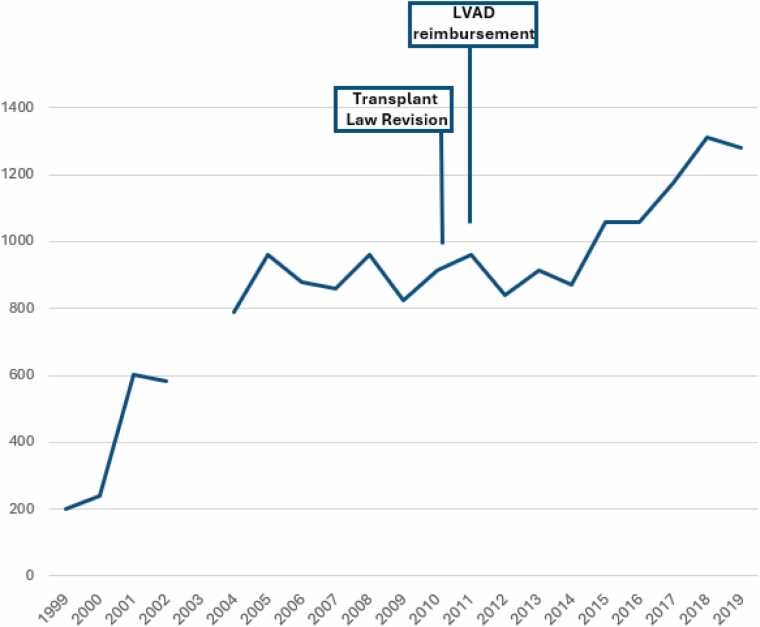
The average wait time (in days) for patients on the heart transplant list in Japan. The prolonged waiting period, extending up to 5 years. LVAD, left ventricular assist device.

**Figure 3:**
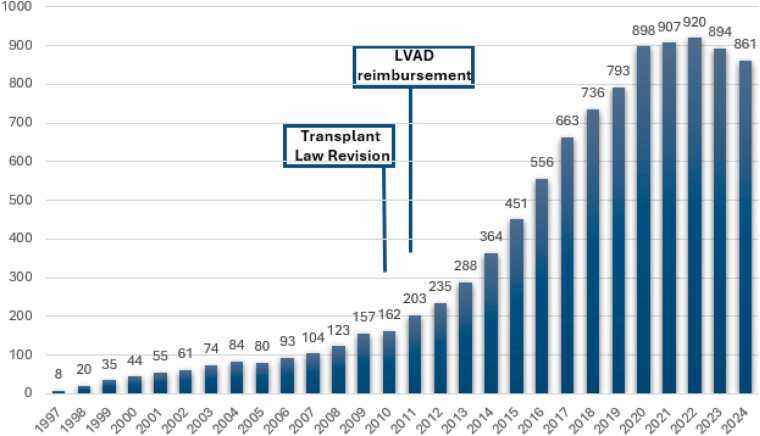
The number of patients listed on the heart transplant list. The number of patients listed on the heart transplant list has gradually increased in the last few decades. LVAD, left ventricular assist device.

This shortfall was further exacerbated by the introduction of durable LVADs in 2011, leading to increased wait times for heart transplants. By 2022, the average wait time for a transplant extended to 1,877 days (Figure 2).^20^ The number of patients on the waitlist escalated to 861 in 2023, a significant rise from just 162 patients in 2010 (Figure 3).^20^ Given the lengthy wait times and the current listing criteria prioritizing patients with durable LVADs as status 1, the majority of transplant recipients are those supported by these devices at the time of transplantation.^20^

Several factors contribute to organ donation shortage, with cultural beliefs might be playing a role. The concept of “kegare” (pollution), rooted in Japan's oldest chronicle, the Kojiki, suggests that death brings contamination that is both polluting and contagious, necessitating avoidance and purification.^21^ The concept of “kegare” might be deeply embedded in Japanese culture and daily life, making it difficult to determine precisely how much it contributes to the community's reluctance to discuss organ donation. A national survey in 2021, involving 1,705 respondents, revealed that only 39.5% had agreed to donate organs.^22^ The primary reasons cited for this hesitancy included a reluctance to donate organs (27%) and fear of organ donation (34%). Moreover, 56% of respondents reported never having discussed organ donation with their family members. Despite efforts by the Japanese government to promote organ donation through media campaigns, these cultural barriers remain.

Medical professionals also face challenges in initiating organ donation discussions, particularly due to the cultural background. However, other systemic issues compound the problem. A critical hurdle is the lack of facilities equipped to evaluate brain death.^23^ Of the 896 hospitals registered to conduct brain death assessments, only 49% are capable of performing such evaluations. Furthermore, of the 435 facilities with the necessary capabilities, only 194 have facilitated organ donation in the past, and 90 of these have done so only once. The process of determining brain death is complex and places a significant burden on primary care providers to initiate discussions about organ donation with the families of potential donors. This is particularly challenging in a culture that is hesitant about organ donation, coupled with providers' unfamiliarity with the process and the heavy workloads they already face. Overwork, a well-recognized issue in Japan limits health care providers' time and energy for engaging in these critical conversations.^24^ To address these challenges, a medical consultant system, established in 2002 to address these challenges, aids in brain death determination, but greater consultant involvement is needed to ease primary providers’ burden and increase organ donations.^25^

In addition, the Japan Organ Transplant Network plays a pivotal role in managing the organ donation process in Japan by overseeing the allocation, recovery, and transport of donor organs.^26^ This centralized system ensures equity and transparency in organ distribution; however, it also presents significant operational challenges, particularly in addressing the critical disparity between the demand and supply of heart donations. One of the primary challenges is the persistent shortage of transplant coordinators, which frequently results in delays in organ evaluation and allocation. Such delays are especially pronounced in geographically remote regions, where logistical hurdles, such as limited transportation infrastructure, further complicate the timely processes of organ donation. Moreover, while Japan Organ Transplant Network’s centralized management approach promotes standardized practices, it can simultaneously reduce the system's adaptability in responding to urgent or unforeseen circumstances. The heavy workload borne by transplant coordinators has been identified as a key factor contributing to operational inefficiencies, potentially compromising the viability of donor organs.^26^

The geographic distribution of organ donors and recipients in Japan adds another layer of complexity to the already critical imbalance between the demand and supply of heart donations. While Japan's universal health care system ensures equal access to basic medical care in principle, significant disparities persist in advanced therapies such as heart transplantation and durable LVADs due to the geographic concentration of specialized centers. With only 11 facilities nationwide performing heart transplants, patients in rural or remote areas face delays in care, financial burdens, and logistical challenges, including the need to relocate. These geographic disparities, combined with limited experience among general cardiologists and surgeons, may hinder the evaluation and management of advanced heart failure patients.

## Challenges to expanding the donor pool in Japan

To facilitate the expansion of the donor pool in Japan, it is imperative to examine and potentially adapt the successful strategies employed in the United States, which have demonstrably broadened the donor base through innovative practices in organ donation and transplantation protocols.

Significant progress has been made in expanding the donor pool for heart transplantation in the United States through key developments.^27^, ^28^ The opioid crisis, which has intensified since the early 2000s, has significantly increased the availability of organ donors. Analysis of Organ Procurement and Transplantation Network data from 2000-2017 shows that the proportion of adult donors who succumbed to drug overdoses and whose hearts were recovered for transplantation increased from 1.1% to 13.4%.^29^ In addition, the introduction of direct-acting antivirals for the treatment of hepatitis C virus (HCV) has allowed many transplant centers to accept organs from HCV-infected donors.^28^ These treatments, characterized by cure rates exceeding 95% with short-course, well-tolerated oral regimens, have led to an increase in the proportion of HCV-positive donors from 0.6% to 11.5% of all heart transplants between 2016 and 2019. Donation after circulatory death (DCD) has also made a significant contribution to expanding the donor pool.^27^, ^28^ A single center in the United Kingdom reported 79 heart transplants from DCD donors between 2015 and 2020, increasing their total heart transplant activity by 48%.^30^ These data illustrate the critical role of DCD donors in increasing the availability of donor hearts for transplantation.

Conversely, Japan faces unique challenges in implementing these strategies due to differences in medical system dynamics and cultural contexts. The relatively low incidence of substance abuse and HCV among Japan's young population, potential donors, limits the applicability of strategies that have been successful in other countries.^31^, ^32^ Significant cultural and legal barriers also affect the use of DCD. Japanese physicians are often reluctant to withdraw care due to legal and cultural factors. The 1968 case of Dr Juro Wada and a 1998 trial at Kawasaki Kyodo Hospital, where a physician was convicted for terminal extubation without explicit consent, have profoundly shaped Japan’s end-of-life care approach.^33^ These cases highlight the legal risks and uncertainties that physicians face in making decisions to withdraw life-sustaining treatment, resulting in a reluctance to discuss or pursue palliative care options.

In addition to these barriers, the adoption of newer technologies, such as normothermic regional perfusion and ex vivo organ perfusion, presents both opportunities and challenges for Japan. In the United States, these advanced techniques have improved graft quality and organ preservation, enabling the procurement of organs from more remote locations and expanding the donor pool.^34^, ^35^ In Japan, while these technologies may hold promise, their actual impact on donor pool expansion is uncertain. Given the country's relatively compact geography and efficient transportation infrastructure, ischemic times are already kept within reasonable limits in most cases. As a result, the extent to which these technologies would significantly expand the donor pool in Japan remains questionable. Moreover, heart transplantation is already associated with high medical costs in Japan, and the integration of technologies such as ex vivo organ perfusion could further escalate these expenses. Careful evaluation will be required to balance the potential benefits of these innovations against their economic impact, particularly in the context of Japan's health care system and its unique donor landscape.

Last, an opt-out organ donation policy, in which individuals are presumed willing donors unless they opt out, has the potential to significantly expand the donor pool and address organ shortages. This approach has been adopted in many countries as a promising strategy, but Japan faces unique legal and sociocultural challenges in implementing such a system.^36^

Legally, transitioning to an opt-out policy would require significant amendments to Japan’s Organ Transplant Act, which currently emphasizes explicit, informed consent. This shift could face constitutional scrutiny under Article 13, which protects individual rights and autonomy. Effective implementation would necessitate public education campaigns and a transparent opt-out registry to ensure awareness and accessibility. Addressing liability concerns for health care providers would also be essential to minimize resistance within the medical community.

Socioculturally, an opt-out system may conflict with Japan’s cultural emphasis on familial decision-making and beliefs about death and bodily integrity. Families may perceive the system as diminishing their role, leading to potential skepticism. Additionally, past controversies surrounding organ transplantation in Japan could heighten public skepticism. These factors highlight the need for comprehensive public engagement and culturally sensitive education to gain acceptance for such a policy.

## Re-evaluating Japan's heart transplantation allocation system: Need for adaptation to support acutely ill patients

Japan's heart transplant allocation system, as detailed in Table 1 and guided by a 2016 expert statement, does not adequately address the needs of the current clinical landscape, particularly for patients with acute cardiogenic shock or those supported by temporary mechanical circulatory devices such as Impella.^37^ This limitation arises from the system's challenges in distinguishing the urgent needs of acutely ill patients from those stabilized with durable LVADs, despite significant differences in their clinical prognoses. Currently, most patients listed in the allocation system are categorized as status 1 and are supported by durable LVADs. Given the average wait time of 5 years and priority determined by wait time, patients with acute cardiogenic shock are unlikely to benefit from heart transplantation, although patients assisted with temporary mechanical circulatory supports could be listed under the same criteria as those with durable LVADs. In Japan, the use of temporary mechanical circulatory support is on the rise, particularly the use of the Impella system, approved in 2016.^38^ While the use of mechanical support is increasing, clinical outcomes remain poor. A retrospective study of 593 patients with cardiogenic shock due to acute myocardial infarction who were supported with Impella showed mortality rates for patients assisted with Impella and ECPELLA were 19.1% and 54.3%, respectively, emphasizing the critical need for sophisticated exit strategies.^39^

**Table 1:** Overview of the Priority Criteria for Organ Transplant Recipients in Japan and the United States

Japanese criteria (2016-present)	United States criteria (2018-present)
Priority | criterion | description	
1. *Family members*	
According to the provision of Article 6-2 of the Organ Transplant Law, family members are prioritized if there is an expressed intention to donate organs to them.	
2. *Medical urgency*	1. *Medical urgency*
*Status 1*: Patients in any of the following conditions:	*Status 1*: ECMO (up to 7 days)
(A) Supported with a ventricular assist device	(A) Nondischargeable, surgically implanted, nonendovascular BiV support device
(B) Undergoing intra-aortic balloon pumping, percutaneous cardiopulmonary support, or veno-arterial bypass	(B) MCS with life-threatening ventricular arrhythmia
(C) On mechanical ventilation	
(D) Being admitted and receiving continuous infusion of inotropes, including phosphodiesterase inhibitors	
*Status 2*: Patients not in Status 1 conditions	*Status 2*
	(A) Nondischargeable, surgically implanted, nonendovascular LVAD (up to 14 days)
	(B) IABP (up to 14 days)
	(C) VT/VF, mechanical support not required
	(D) MCS with device malfunction/mechanical failure
	(E) TAH, BiVAD, RVAD, or VAD for single ventricle patients
	(F) Percutaneous endovascular MCS (up to 14 days)
*Status 3*: Temporarily removed from the waiting list due to disqualifying conditions (e.g., infections) but can be relisted once these conditions are resolved. Prioritization follows from Status 1 to Status 2, with Status 3 being excluded until requalification	*Status 3*
	(A) Dischargeable LVAD for discretionary 30 days
	(B) Multiple inotropes or single high-dose inotropes with continuous hemodynamic monitoring
	(C) MCS with device infection, hemolysis, pump thrombosis, right heart failure, mucosal bleeding, or aortic insufficiency
	(D) Temporary MCS after 14 days (7 days for ECMO) without reapproval
	*Status 4*
	(A) Stable LVAD candidates not using 30-day discretionary period
	(B) Inotropes without hemodynamic monitoring
	(C) CHD, HCM/RCM, or amyloidosis
	(D) IHD with intractable angina
	(E) Retransplantation
	*Status 5*
	(A) Combined organ transplants (on waitlist for at least 1 other organ at same hospital)
	*Status 6*
	(A) All remaining active candidates
3. *Age*	
Priority is determined based on the age of the donor and the age of the recipient at the time of registration with the Japan Organ Transplant Network, according to specific selection criteria.	
4. *Blood type*	
Recipients with an identical ABO blood type to the donor are prioritized over those who are merely compatible.	
5. *Waiting period*	
Among recipients with identical conditions, those who have been waiting longer are prioritized. For Status 1 recipients, the waiting period counts the total days in Status 1.	
Note: For patients registered under 18 and in Status 1 due to condition (D), who then age over 18 and are not in an ICU/CCU and thus categorized as Status 2, but later return to a Status 1 condition, the waiting period during their initial Status 1 is excluded. For Status 2 recipients, the waiting period is calculated from the registration date.	

Abbreviations: BiV, biventricular; BiVAD, biventricular assist device; CHD, congenital heart disease; ECMO, extracorporeal membrane oxygenation; HCM, hypertrophic cardiomyopathy; HLA, human leukocyte antigen; IABP, intra-aortic balloon pump; IHD, ischemic heart disease; LVAD, left ventricular assist device; MCS, mechanical circulatory support; RCM, restrictive cardiomyopathy; RVAD, right ventricular assist device; TAH, total artificial heart; VAD, ventricular assist device; VF, ventricular fibrillation; VT, ventricular tachycardia.

In Japan, organ transplant prioritization is determined by factors including familial consent, medical urgency, patient age, blood type, and waiting period, as specified by the Organ Transplant Law.

The introduction of these devices likely prolonged survival; however, it has led to challenges among providers due to limited available exit strategies for these patients. Fortunately, durable LVADs have become widely available across the country. At present, 45 hospitals are registered to implant durable LVADs, and implantation is feasible if the patient is transportable.^40^ However, if patients have any contraindications for durable LVAD, such as severe right ventricular dysfunction or a small left ventricular cavity, the only options are prolonged optimistic waiting for myocardial recovery or transition to palliative care, which is less likely in the context of Japan's complicated history with palliative medicine.^33^

In Japan, the survival rates for durable LVADs, predominantly HeartMate 3, are excellent, with 1-, 2-, and 3-year survival rates at 94%, 92%, and 88%, respectively.^14^ These outcomes are comparable to those reported in the MOMENTUM 3 trial, which demonstrated a 1-year survival rate of 82.8% and a 2-year survival rate of 79.5% for HeartMate 3 recipients.^41^ The higher survival rates in Japan may reflect differences in patient demographics, as Japanese LVAD recipients tend to be younger and have fewer comorbidities compared to those in the MOMENTUM 3 trial.

Given these favorable outcomes for LVAD patients in Japan, it may be reasonable to consider prioritizing donor hearts for patients with acute cardiogenic shock who require urgent transplantation, particularly for those who are not candidates for LVAD implantation due to conditions such as severe right ventricular dysfunction or a small left ventricular cavity. However, this approach raises significant ethical challenges, as it would involve deprioritizing patients who have been waiting for years on the transplant list under the current system. Balancing the need to address the urgency of acute cases with the ethical obligation to patients who have endured prolonged wait times remains a complex and contentious issue. The immediate task is to carefully consider the management of patients with cardiogenic shock and to adjust the priority of patients with LVAD complications, similar to the revision of the allocation system in the United States.^42^

The prioritization of donor hearts for acutely ill patients with cardiogenic shock is a separate issue that needs to be discussed independently from the prioritization of patients with LVADs. This model has already been adopted in the United States. In 2018, United Network for organ sharing implemented a 6-tier allocation policy, replacing the previous 3-tier system (Table 1).^42^ This policy, which responded to the growing history list of critically ill heart transplant candidates and extended waitlist times, aimed to better stratify candidates by waitlist mortality, reduce wait times for high-priority candidates, and address disparities. These adjustments have resulted in shorter wait times and improved survival on the waiting list.

Similarly, in Europe, the International High Urgency Status is granted to patients with critical conditions, such as those hospitalized in intensive care units receiving inotropic support with hemodynamic monitoring, exhibiting early signs of secondary organ failure, experiencing complications or failure of a mechanical assist device, or requiring acute retransplantation due to primary graft failure within the first week post-transplantation.^43^ In addition to these international criteria, each European country has its own national guidelines, which may vary slightly. For instance, in 2018, France introduced a national scoring system ranging from 0 to 1,151, where higher scores correspond to a greater likelihood of receiving a heart transplant.^44^ Despite these regional variations, the commonality among European countries, as well as the United States, is the prioritization of acutely ill patients—contrasting with Japan's approach.

## The urgent need for advanced heart failure specialist training

The expected increase in the number of patients with heart failure is expected to increase the demand for advanced therapies. This projected increase is likely to result in an increase in the number of patients admitted to cardiac intensive care units who are in cardiogenic shock, require mechanical circulatory support, and face complications associated with heart transplantation and durable LVADs in the near future. In the United States, the Heart Failure Society of America, recognizing similar challenges, emphasized the importance of establishing board certification and fellowship programs for advanced heart failure and transplant cardiologists in 2008.^45^ In 2013, a formal training and certification pathway for Advanced Heart Failure and Transplant Cardiology was officially established, with the goal of training specialists to treat patients who require advanced therapies and manage the resulting complications. This standardization will ensure that all cardiologists have a comprehensive understanding of advanced therapies, facilitating timely and appropriate referral to specialists in advanced heart failure and transplant cardiology.^46^

**Figure 4:**
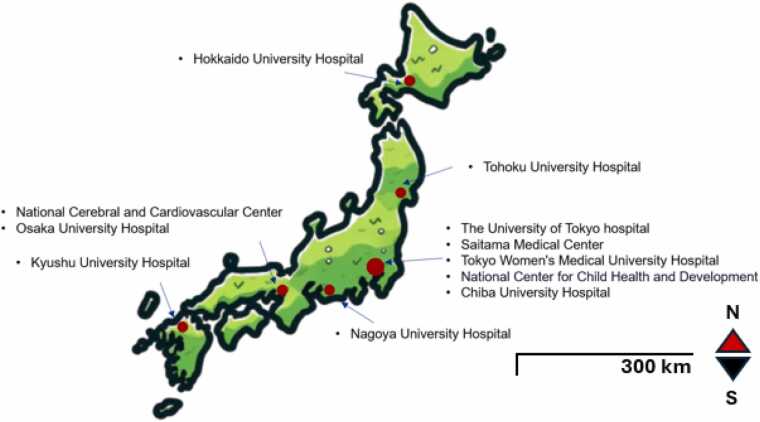
Heart transplant facilities in Japan. In Japan, 11 facilities are registered to perform heart transplantation.

In contrast, Japan lacks a board certification for heart failure and a formalized education framework for cardiologists to become acquainted with advanced therapies. Such treatments are confined to a select few centers, with only 11 facilities nationwide registered to perform heart transplants (Figure 4).^13^ Despite the opportunity for advanced training in experienced centers, concerns remain, particularly regarding inadequate exposure to heart transplantation. Notably, only 2 centers in Japan have managed to surpass an annual transplant volume of 20 cases, despite a relatively homogeneous pretransplant population predominantly supported by durable LVADs and characterized by minimal medical comorbidities and younger age demographics.^47^ Moreover, the relatively recent adoption of heart transplantation in Japan has ignited ongoing debates over the management of older transplant recipients. It is imperative to develop a medical education system that prepares general cardiologists with knowledge of advanced heart failure but also specializes in training for advanced heart failure and transplant cardiologists to prevent overwhelming cardiac intensive care units.

Additionally, establishing training programs for intensivists specializing in critical care cardiology is crucial, especially considering the projected increase in noncardiac admissions (e.g., sepsis, respiratory failure, acute kidney injury) as heart failure prevalence rises.^48^, ^49^ The availability of such specialists is linked to improved clinical outcomes.^50^, ^51^, ^52^ However, Japan faces a significant shortage of intensivists, particularly those with medical training. As of April 1, 2023, only 2,550 intensive care specialists have been certified by the Japanese Society of Intensive Care Medicine, representing a mere 0.75% of the country's total physician workforce of approximately 340,000.^53^, ^54^ The Japanese Society of Intensive Care Medicine’s report further highlights this shortfall, indicating that the current number of intensive care specialists meets only about 30% of the demand necessary for optimizing patient outcomes, thereby highlighting the critical need for more specialists in this area.^55^

## Global lessons from Japan: Advancing heart failure care

Insights from the implementation of advanced heart failure care in Japan offer valuable lessons for other countries navigating similar challenges. For example, the widespread adoption of LVADs in Japan has contributed to a substantial increase in the number of patients awaiting heart transplantation. This situation highlights the need for countries to anticipate and address imbalances between the demand for transplants and the availability of donor organs through strategic planning and policy adjustments.

Cultural and societal factors, deeply rooted in traditions and beliefs, play a pivotal role in shaping attitudes toward organ donation. In Japan, concerns related to bodily integrity after death can pose significant barriers to donor registration. This underscores the importance of designing public education campaigns and policy interventions that are informed by the cultural context, ensuring that they resonate with the population and promote greater acceptance of organ donation.

As more patients access advanced therapies, the demand for highly trained specialists continues to grow. The Japanese health care system exemplifies the critical importance of proactive investment in medical education. Establishing robust training programs and standardized pathways for certification can prepare the workforce to meet the increasing complexity of advanced heart failure care, a challenge shared by many nations.

Efforts to expand the donor pool in Japan also provide a valuable framework for global application. Practical approaches, such as raising public awareness and implementing policies that address systemic barriers, must be tailored to each country's unique demographic and cultural landscape. These strategies can serve as a blueprint for overcoming similar obstacles in other health care systems.

By reflecting on the experiences in Japan, countries worldwide can develop sustainable models for advanced heart failure care and transplantation. This includes addressing cultural, systemic, and workforce challenges while ensuring equitable access to life-saving therapies.

## Conclusion

Our study highlights the challenges of advanced heart failure in Japan. Despite advances in treatment and legislative efforts to improve organ donation, significant obstacles remain, including cultural and policy barriers, a shortage of organ donors, and gaps in continuing medical education. It is critical to expand the donor pool, reform the heart transplant allocation system, and strengthen specialized training programs. Overcoming these challenges will require a comprehensive strategy that integrates cultural sensitivity, legislative changes—including the 2010 amendment to the Organ Transplant Act, prioritizing sicker patients in the heart transplant allocation system, considering an opt-out organ donation policy, and improving the brain death determination process—and global best practices to improve outcomes for heart failure patients in Japan.

## Author Contributions

Satoshi Miyashita, MD: Conceived the study, led the writing and revisions of the manuscript, and is the corresponding author, ensuring accountability for all aspects of the work. Francisco B. Alexandrino, MD: Contributed to the design, and interpretation of data; participated in drafting and critically revising the manuscript. Amanda Vest, MBBS, MD, MPH: Contributed to the design, and interpretation of data; participated in drafting and critically revising the manuscript. Yasumasa Tsukamoto, MD, PhD and Tomohiro Fujisaki, MD: Assisted in data interpretation and critically reviewed the manuscript for intellectual content. Koichiro Kinugawa, MD, PhD: Contributed to the manuscript's conception, design, and critical revision process, and provided final approval of the version to be published.

## Disclosure statement

None.

The author confirms that the work described in this article has not been published previously. This article is not under consideration for publication elsewhere, and its publication is approved by all authors and authorized by the responsible institutions where the work was conducted. If accepted, this article will not be published in the same form, in English or any other language, including electronically, without the written consent of the copyright holder.

## References

[1] Bozkurt B., Ahmad T., Alexander K.M. (2023). Heart failure epidemiology and outcomes statistics: a report of the Heart Failure Society of America. J Card Fail.

[2] Savarese G., Becher P.M., Lund L.H., Seferovic P., Rosano G.M.C., Coats A.J.S. (2023). Global burden of heart failure: a comprehensive and updated review of epidemiology. Cardiovasc Res.

[3] Ministry of Health, Labour, and Welfare; July 2022. Available at: https://www.mhlw.go.jp/toukei/saikin/hw/life/life10/, accessed February 21, 2024.

[4] Isobe M. (2019). The heart failure “pandemic” in Japan: reconstruction of health care system in the highly aged society. JMA J.

[5] Ohira T., Eguchi E., Hayashi F., Kinuta M., Imano H. (2024). Epidemiology of cardiovascular disease in Japan: an overview study. J Cardiol.

[6] The results of 2019 national health and nutritional investigation; October 2020. Available at: https://www.mhlw.go.jp/stf/newpage_14156.html, accessed February 21, 2024.

[7] Zhang L., Ono Y., Qiao Q. (2023). Trends in heart failure prevalence in Japan 2014–2019: a report from healthcare administration databases. ESC Heart Fail.

[8] Shiraishi Y., Kohsaka S., Sato N. (2018). 9-year trend in the management of acute heart failure in Japan: a report from the national consortium of acute heart failure registries. J Am Heart Assoc.

[9] Heidenreich P.A., Bozkurt B., Aguilar D. (2022). 2022 AHA/ACC/HFSA guideline for the management of heart failure: a report of the American College of Cardiology/American Heart Association joint committee on clinical practice guidelines. Circulation.

[10] Nishimoto Y., Ohbe H., Matsui H. (2023). Trends in mechanical circulatory support use and outcomes of patients with cardiogenic shock in Japan, 2010 to 2020 (from a Nationwide Inpatient Database Study). Am J Cardiol.

[11] Jorde U.P., Saeed O., Koehl D. (2024). The Society of Thoracic Surgeons Intermacs 2023 annual report: focus on magnetically levitated devices. Ann Thorac Surg.

[12] OPTN Metrics; February 2024. Available at: https://insights.unos.org/OPTN-metrics/, accessed February 21, 2024.

[13] JOT; February 2024. Available at: https://www.jotnw.or.jp/data/offer03.php, accessed February 21, 2024.

[14] Japanese Registry for Mechanically Assisted Circulatory Support J-MACS Statistical Report; April 2023. Available at: https://j-vad.jp/jmacs-report/, accessed February 21, 2024.

[15] Toshima H., Kawai C. (1995). Why is heart transplantation not performed in Japan? Refutation of Dr Yoshio Watanabe's arguments against heart transplantation. Jpn Heart J.

[16] Kinugawa K., Sakata Y., Ono M. (2021). Consensus report on destination therapy in Japan ? From the DT committee of the council for clinical use of ventricular assist device related academic societies. Circ J.

[17] Feng J., Zhang Y., Zhang J. (2024). Epidemiology and burden of heart failure in Asia. JACC Asia.

[18] Bates B.A., Enzan N., Tohyama T. (2024). Management and outcomes of heart failure hospitalization among older adults in the United States and Japan. ESC Heart Fail.

[19] IRODAT; 2022. Available at: https://www.irodat.org/?p=database, accessed February 21, 2024.

[20] The Japanese Society for Heart Transplantation (2023). The registry report of Japanese heart transplantation-2023. Jpn J Transplant.

[21] Asai A., Masaki S., Okita T., Enzo A., Kadooka Y. (2018). Matters to address prior to introducing new life support technology in Japan: three serious ethical concerns related to the use of left ventricular assist devices as destination therapy and suggested policies to deal with them. BMC Med Ethics.

[22] Public Opinion Survey on Transplant Medicine; 2021. Available at: https://survey.gov-online.go.jp/r03/r03-ishoku/, accessed February 21, 2024.

[23] Current Status of Organ Transplantation Measures; 2023. Available at: https://www.mhlw.go.jp/content/10900000/001066715.pdf, accessed February 22, 2024.

[24] Koike S., Wada H., Ohde S., Ide H., Taneda K., Tanigawa T. (2024). Working hours of full-time hospital physicians in Japan: a cross-sectional nationwide survey. BMC Public Health.

[25] Fukushima N., Ono M., Saiki Y., Minami M., Konaka S., Ashikari J. (2013). Donor evaluation and management system (medical consultant system) in Japan: Experience from 200 consecutive brain-dead organ donation. Transplant Proc.

[26] Ministry of Health, Labour and Welfare of Japan; 2024. Available at: https://www.mhlw.go.jp/content/10900000/001288160.pdf, accessed December 16, 2024.

[27] Pagani F.D. (2022). Heart transplantation using organs from donors following circulatory death: the journey continues. J Am Coll Cardiol.

[28] DeFilippis E.M., Khush K.K., Farr M.A., Fiedler A., Kilic A., Givertz M.M. (2022). Evolving characteristics of heart transplantation donors and recipients: JACC focus seminar. J Am Coll Cardiol.

[29] Durand C.M., Bowring M.G., Thomas A.G. (2018). The drug overdose epidemic and deceased-donor transplantation in the United States a national registry study. Ann Intern Med.

[30] Messer S., Cernic S., Page A. (2020). A 5-year single-center early experience of heart transplantation from donation after circulatory-determined death donors. J Heart Lung Transplant.

[31] Ko K., Akita T., Satake M., Tanaka J. (2021). Epidemiology of viral hepatitis C: road to elimination in Japan. Glob Health Med.

[32] Yamamoto J. (2004). Recent trends of drug abuse in Japan. Ann N Y Acad Sci.

[33] Makino J., Fujitani S., Twohig B., Krasnica S., Oropello J. (2014). End-of-life considerations in the ICU in Japan: ethical and legal perspectives. J Intensive Care.

[34] Benkert A.R., Keenan J.E., Schroder J.N. (2024). Early U.S. heart transplant experience with normothermic regional perfusion following donation after circulatory death. JACC Heart Fail.

[35] Schroder J.N., Patel C.B., DeVore A.D. (2023). Transplantation outcomes with donor hearts after circulatory death. N Engl J Med.

[36] Jou S., Mendez S.R., Feinman J. (2024). Heart transplantation: advances in expanding the donor pool and xenotransplantation. Nat Rev Cardiol.

[37] Statement for heart transplantation (JCS 2016); 2016. Available at: https://www.j-circ.or.jp/. accessed February 21, 2024.

[38] Nishimoto Y., Ohbe H., Matsui H. (2023). Trends in mechanical circulatory support use and outcomes of patients with cardiogenic shock in Japan, 2010 to 2020 (from a Nationwide Inpatient Database Study). Am J Cardiol.

[39] Ikeda Y., Ako J., Toda K. (2023). Short-term outcomes of Impella support in Japanese patients with cardiogenic shock due to acute myocardial infarction - Japanese registry for percutaneous ventricular assist device (J-PVAD). Circ J.

[40] Council for Clinical Use of Ventricular Assist Device Related Academic Societies; 2024. Available at: https://j-vad.jp/registry-licensed-facilities-adult/, accessed February 21, 2024.

[41] Mehra M.R., Uriel N., Naka Y. (2019). A fully magnetically levitated left ventricular assist device - final report. N Engl J Med.

[42] Maitra N.S., Dugger S.J., Balachandran I.C., Civitello A.B., Khazanie P., Rogers J.G. (2023). Impact of the 2018 UNOS heart transplant policy changes on patient outcomes. JACC Heart Fail.

[43] González-Costello J., Pérez-Blanco A., Delgado-Jiménez J. (2024). Review of the allocation criteria for heart transplant in Spain in 2023. SEC-Heart Failure Association/ONT/SECCE consensus document. Rev Esp Cardiol (Engl Ed).

[44] Dorent R., Jasseron C., Audry B. (2020). New French heart allocation system: comparison with Eurotransplant and US allocation systems. Am J Transplant.

[45] Jessup M., Drazner M.H., Book W. (2017). 2017 ACC/AHA/HFSA/ISHLT/ACP advanced training statement on advanced heart failure and transplant cardiology (revision of the ACCF/AHA/ACP/HFSA/ISHLT 2010 clinical competence statement on management of patients with advanced heart failure and cardiac transplant): a report of the ACC competency management committee. J Am Coll Cardiol.

[46] Jessup M., Ardehali R., Konstam M.A. (2015). COCATS 4 task force 12: training in heart failure. J Am Coll Cardiol.

[47] Oda N., Kato T.S., Komamura K. (2010). Clinical course and outcome of heart transplant recipients single center experience at the National Cardiovascular Center in Japan. Int Heart J.

[48] Bohula E.A., Katz J.N., Van Diepen S. (2019). Demographics, care patterns, and outcomes of patients admitted to cardiac intensive care units: the critical care cardiology trials network prospective North American multicenter registry of cardiac critical illness. JAMA Cardiol.

[49] Grupper A., Chernomordik F., Herscovici R. (2023). The burden of heart failure in cardiac intensive care unit: a prospective 7 years analysis. ESC Heart Fail.

[50] Sims D.B., Kim Y., Kalininskiy A. (2022). Full-time cardiac intensive care unit staffing by heart failure specialists and its association with mortality rates. J Card Fail.

[51] Elliott Miller P., Chouairi F., Thomas A. (2021). Transition from an open to closed staffing model in the cardiac intensive care unit improves clinical outcomes. J Am Heart Assoc.

[52] Kapoor K., Verceles A.C., Netzer G. (2017). A collaborative cardiologist-intensivist management model improves cardiac intensive care unit outcomes. J Am Coll Cardiol.

[53] Overview of the 2020 Statistics for Physicians, Dentists, and Pharmacists; 2020. Available at: https://www.mhlw.go.jp/toukei/saikin/hw/ishi/20/dl/R02_kekka-1.pdf, accessed February 22, 2024.

[54] The Japanese Society of Intensive Care Medicine; 2023. Available at: https://www.jsicm.org/en/, accessed February 22, 2024.

[55] Doi K., Kawai Y., Unogi K. (2022). Recommendations for strengthening the intensive care medical provision system in our country. J Jpn Soc Intensive Care Med.

